# The egg ribonuclease SjCP1412 accelerates liver fibrosis caused by *Schistosoma japonicum* infection involving damage-associated molecular patterns (DAMPs)

**DOI:** 10.1017/S0031182023001361

**Published:** 2024-03

**Authors:** Qi-Feng Li, Yi-Xin Li, Ying-Ying Yang, Pan-Pan Dong, Cong-Jin Mei, Ju-Lu Lu, Jian-Feng Zhang, Hai-Yong Hua, Chun-Rong Xiong, Chuan-Xin Yu, Li-Jun Song, Kun Yang

**Affiliations:** National Health Commission Key Laboratory of Parasitic Disease Control and Prevention, Jiangsu Provincial Key Laboratory on Parasite and Vector Control Technology, Jiangsu Provincial Medical Key Laboratory, Jiangsu Institute of Parasitic Diseases, Wuxi, Jiangsu 214064, China

**Keywords:** DAMPs, egg antigen, liver fibrosis, *Schistosoma japonicum*, SjCP1412

## Abstract

Schistosomiasis, a parasite infectious disease caused by *Schistosoma japonicum*, often leads to egg granuloma and fibrosis due to the inflammatory reaction triggered by egg antigens released in the host liver. This study focuses on the role of the egg antigens CP1412 protein of *S. japonicum* (SjCP1412) with RNase activity in promoting liver fibrosis. In this study, the recombinant egg ribonuclease SjCP1412, which had RNase activity, was successfully prepared. By analysing the serum of the population, it has been proven that the anti-SjCP1412 IgG in the serum of patients with advanced schistosomiasis was moderately correlated with liver fibrosis, and SjCP1412 may be an important antigen associated with liver fibrosis in schistosomiasis. *In vitro*, the rSjCP1412 protein induced the human liver cancer cell line Hep G2 and liver sinusoidal endothelial cells apoptosis and necrosis and the release of proinflammatory damage-associated molecular patterns (DAMPs). In mice infected with schistosomes, rSjCP1412 immunization or antibody neutralization of SjCP1412 activity significantly reduced cell apoptosis and necroptosis in liver tissue, thereby reducing inflammation and liver fibrosis. In summary, the SjCP1412 protein plays a crucial role in promoting liver fibrosis during schistosomiasis through mediating the liver cells apoptosis and necroptosis to release DAMPs inducing an inflammatory reaction. Blocking SjCP1412 activity could inhibit its proapoptotic and necrotic effects and alleviate hepatic fibrosis. These findings suggest that SjCP1412 may be served as a promising drug target for managing liver fibrosis in schistosomiasis japonica.

## Graphical abstract

**Figure:**
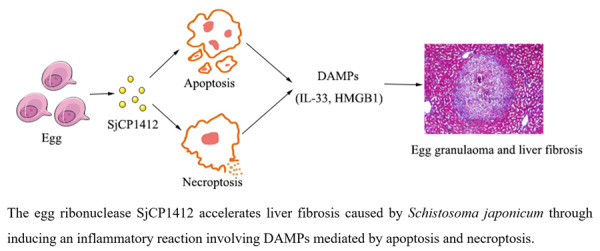

## Introduction

Schistosomiasis is a neglected tropical disease that seriously endangers human health (LoVerde, 2019) and is prevalent in 78 countries worldwide; approximately 800 million people are at risk, and approximately 252 million people suffer from it (Hotez *et al*., 2014). Schistosomiasis causes 280 000 deaths worldwide every year, and 3.8 million disability-adjusted life years are attributed to this disease (McManus *et al*., 2018; Deol *et al*., 2019; Ogongo *et al*., 2022). Chronic schistosome infection can cause liver fibrosis, accompanied by hepatosplenomegaly and portal hypertension, eventually leading to irreversible cirrhosis and liver cancer (Hotez *et al*., 2009). Therefore, it is necessary to understand the formation mechanism of liver fibrosis and develop effective measures to control liver fibrosis.

There are 3 main species infecting humans: *Schistosoma mansoni* (*S. mansoni*), *Schistosoma japonicum* (*S. japonicum*) and *Schistosoma haematobium* (*S. haematobium*). Eggs of *S. mansoni* and *S. japonicum*, laid by adult worms and retained in the liver, continue to produce secretions known as soluble egg antigen (SEA), inducing the host to produce an inflammatory response. Monocytes, eosinophils, macrophages and other cells enwrap the eggs to form granulomas (Booth *et al*., 2004; Burke *et al*., 2009; Fairfax *et al*., 2012). Many cytokines and chemokines stimulate quiescent hepatic stellate cells to transform into activated hepatic stellate cells, which secrete excessive extracellular matrix and lead to the development of fibrosis (Krenkel and Tacke, 2017; Tacke, 2017).

Among many factors that lead to liver fibrosis, cell death including apoptosis and necrosis is the initiating factor, which has been reported in other liver diseases (Luedde *et al*., 2014; Seki and Schwabe, 2015). Apoptosis is a highly regulated mode of cell death mediated by the caspase cascade, which is the most common mode of cell death. During this process, apoptotic bodies are formed by the plasma membrane, blocking the release of cellular contents (Elmore, 2007). Some kinases and transcription factors are inactivated by caspase, thereby inhibiting the production and secretion of inflammatory cytokines (Fischer *et al*., 2003). Although apoptosis is considered an immune silencing process, apoptotic cells can release damage-associated molecular patterns (DAMPs) into the surroundings in response to external stimulation or intracellular pathogens, such as high mobility group protein 1 (HMGB1), calreticulin, heat shock protein 90, adenine nucleoside triphosphate, uric acid, DNA fragments, mitochondrial contents and some cytokines, such as interleukin (IL)-1*β* and IL-33, which are involved in the inflammatory response (Garg *et al*., 2012; Kaczmarek *et al*., 2013). Necroptosis is a regulated form of necrosis that has the same upstream molecular mechanism as apoptosis but has a necrotic phenotype, which is characterized by organelle and cell swelling, cell membrane rupture leading to the leakage of cellular contents, the release of DAMPs and the promotion of the inflammatory response (Schwabe and Luedde, 2018; Fritsch *et al*., 2019). Our previous results showed that (Song *et al*., 2022) the marker proteins of apoptosis and necroptosis were increased after infection with schistosomes and showed a positive correlation with the pathological damage of liver fibrosis, indicating that cell death is involved in the development of liver fibrosis induced by schistosomes. However, it is currently unclear how cell death is caused during schistosomiasis and which antigen component is involved in cell death.

In a previous study, we analysed the antigens of *S. japonicum* eggs and found that SjCP1412 (GenBank: AY570741.2) was a unique RNase in the exocrine proteins specifically expressed in *S. japonicum* eggs (Ke *et al*., 2017). SjCP1412 has a conserved functional domain similar to omega-1 (GenBank: Smp_193860), a hepatotoxic molecule in *S. mansoni* eggs (Doenhoff *et al*., 1981; Dunne *et al*., 1991) and belongs to the RNase T2 family (Ke *et al*., 2017), which could degrade RNA and may cause cell death. In this study, the recombinant SjCP1412 (rSjCP1412) protein was expressed in *Escherichia coli* BL21(DE3) and purified with Ni-NTA affinity chromatography column. The correlation between anti-rSjCP1412 IgG and liver fibrosis in advanced schistosomiasis patients was analysed using Spearman rank correlation. Human liver cancer cell lines (Hep G2) and liver sinusoidal endothelial cells (LSECs) were stimulated with different concentrations of rSjCP1412 for 48 h, the proliferation was detected with the cell counting kit-8 (CCK-8). The apoptosis and necrosis were tested with Annexin V-FITC/PI double staining by flow cytometry. The levels of DAMPs in the supernatant were detected with enzyme-linked immunosorbent assay (ELISA). Then the effects of rSjCP1412 immunization or neutralization of anti-SjCP1412 protein IgG on egg granuloma and liver fibrosis in mice were observed.

## Materials and methods

### Cells, snails and animals

The human liver cancer cell line Hep G2 was purchased from Procell Life Science & Technology Co., Ltd., and human LSECs were purchased from Wanwu Biotechnology Co., Ltd. The cells were cultured in DMEM supplemented with 10% FBS and 1% penicillin/streptomycin. Infected snails came from the Department of Snail Biology of the Jiangsu Institute of Parasitic Diseases. Female BALB/c (6–8 weeks old, 15–25 g, SPF) mice were purchased from the Animal Experiment Centre of Yangzhou University. The male rabbits (6 months old, weighing 2–3 kg) were obtained from the Jinling Animal Centre (Nanjing, China).

### Sera from patients and healthy individuals

Forty-five serum samples of advanced schistosomiasis patients were collected from local hospitals in Changshu City, Suzhou City, Baoying County, Gaoyou and Jiangdu District, Yangzhou, China. Thirty serum samples were collected from healthy individuals at the Heath Management Centre of the Jiangsu Institute of Parasitic Diseases.

### Recombinant SjCP1412 protein expression and purification

The gene encoding SjCP1412 was synthesized by Shanghai Jierui Biological Co., Ltd. and cloned into the pET28b (+) expression plasmid with Nco1 and Xho1 double restriction enzyme sites to form the recombinant pET28b-SjCP1412 expression plasmid. Then, the pET28b-SjCP1412 recombinant plasmid was transformed into *E. coli* BL21 (DE3) to construct the genetically engineered strain pET28b-SjCP1412/*E. coli* BL21 (DE3), which was induced with 0.5 mmol L^−1^ IPTG to express recombinant SjCP1412 (rSjCP1412). rSjCP1412 was purified with denatured nickel-affinity chromatography and refolded with a gradient urea solution containing 0.5 mol L^−1^ arginine. The endotoxin was removed from the purified rSjCP1412 by the Toxin Eraser TM endotoxin removal kit (Genscript, China), and the purified protein was used for the subsequent experiments.

### Ribonuclease activity of rSjCP1412

A total of 200 ng of yeast RNA was incubated with 10, 20 and 40 *μ*g of rSjCP1412 at 37°C for 2 h, respectively. The same amount of yeast RNA digested with 10 *μ*g of RNase A and undigested yeast RNA were used as positive and negative controls, respectively. The hydrolysate was subjected to 1.5% agarose gel electrophoresis to observe the ribonuclease activity of rSjCP1412.

### Analysis of anti-rSjCP1412 and SEA IgG levels in the serum of advanced schistosomiasis and healthy subjects

SEA was prepared as previously described (Boros and Warren, 1970), endotoxin was removed by the Toxin Eraser TM endotoxin removal kit (Genscript, China), and 1 *μ*g of rSjCP1412 was coated on the surface of per well of microplate and incubated at 4°C overnight. Five percent skimmed milk powder was used to block the samples at 37°C for 2 h. Patient serum (1:50) was incubated at 37°C for 1 h. Goat anti-human IgG (Proteintech, China) was incubated at 37°C for 1 h, and TMB was used for development. The absorbance at 450 nm was measured with a microplate reader (Molecular Device, USA).

### Cell proliferation assay

A total of 1 × 10^4^ Hep G2 cells or LSECs were transferred onto a 96-well plate. After 24 h, rSjCP1412 or SEA at final concentrations of 12.5, 25.0 and 50.0 *μ*g mL^−1^ was added to each well, respectively, to stimulate cell proliferation. Wells containing culture medium, CCK-8 solution alone and rSjCP1412 were used as the blank. Wells containing cells, culture medium and CCK-8 solution without rSjCP1412 were used as the control. Ten microlitres of CCK-8 solution (MCE, China) was added to each well after further incubation for 48 h. After 45 min, the A450 value was measured using a microplate reader (Molecular Device, USA).

### Analysis of cell death by flow cytometry

A total of 1 × 10^5^ Hep G2 cells or LSECs were cultured in a 6-well plate for 24 h. rSjCP1412 (12.5, 25.0 and 50.0 *μ*g mL^−1^) and SEA (25 *μ*g mL^−1^) were added, respectively. After 48 h, the Annexin V-FITC Apoptosis Detection Kit (BD, USA) was used to detect apoptosis and necrosis by flow cytometry (BD Verse, USA). Early apoptotic cells were Annexin V-FITC (+) and PI (−). Late apoptotic cells or necrotic cells were Annexin V-FITC (+) and PI (+). Mechanical necrotic cells and non-specific cells were Annexin V-FITC (−) and PI (+). Surviving cells were Annexin V-FITC (−) and PI (−) (Park *et al*., 2019; Kari *et al*., 2022).

### Preparation of anti-rSjCP1412 polyclonal antibodies

Two rabbits were subcutaneously immunized with 1 mL of complete Freund's adjuvant antigen (Sigma, USA) containing 1 mg of rSjCP1412 in the neck and back intracutaneously. Then, the same dose of incomplete Freund's adjuvant antigen (Sigma) containing 1 mg of rSjCP1412 was used to boost the immunization 2 times every 2 weeks. rSjCP1412 (0.5 mg) in PBS was intramuscularly injected into the leg. Blood was collected through the ear vein before the first immunization and after the last immunization, and the antibody titre in the serum was determined by ELISA as described above. Polyclonal antibodies against rSjCP1412 were purified using an affinity chromatography column combined with rSjCP1412.

### Establishment of liver fibrosis in mice infected with *S. japonicum*

Twenty-four BALB/c mice were randomly divided into 3 groups: the infection group, the immune group and the antibody neutralization group. Each group included 8 mice. Mice in the immunization group were intracutaneously immunized with 100 *μ*L of complete Freund's adjuvant antigen containing 50 *μ*g of rSjCP1412. Subsequently, booster immunizations were performed every 2 weeks with incomplete Freund's adjuvant antigen containing 50 *μ*g of rSjCP1412, and each mouse was infected with 15 ± 2 cercariae after the final immunization. In the neutralization group, each mouse was infected with 15 ± 2 cercariae, and then 10 *μ*g/g purified anti-rSjCP1412 IgG was intraperitoneally injected at 3rd week first after infection, followed by injections every 3 days until the 8th week post infection. A total of 10 times injections have been performed. The mice were sacrificed and perfused at 8th week after infection, and the number of adult worms was counted. The liver was digested with 5% KOH, and the number of eggs was counted. Blood was collected from the inner canthal orbital vein, and serum was isolated and stored at −80°C. The spleen was weighed, and the spleen index (SI) was calculated as follows: SI = (spleen weight/mouse weight) × 10.

### ELISA analysis of DAMPs levels in human serum, mouse serum and cell culture supernatants

The levels of DAMPs (IL-33, IL-1*β* and HMGB1) in human serum, mouse serum and cell cultures were detected with an ELISA detection kit (Multi Sciences, China) according to the manufacturer's instructions.

### Analysis of serum alanine transaminase (ALT) and aspartate aminotransferase (AST) levels in mice

The levels of ALT and AST in mouse serum were detected using kits from Nanjing Jiancheng Biology Co., Ltd. (China).

### Hydroxyproline levels in mouse livers

The hydroxyproline levels in mouse livers were detected using a kit from Nanjing Jiancheng Biological Co., Ltd.

### Haematoxylin-eosin (HE) and Masson staining

The liver tissue was fixed in 4% paraformaldehyde, dehydrated and embedded. Slices with a thickness of 4 *μ*m were dewaxed in water, stained with HE and Masson detection kits from Solarbio Science & Technology Co., and photographed under a microscope (Olympus, Japan). The mean areas of the single egg granuloma and collagen were calculated with cellSens (Olympus, Japan).

### Fluorescence quantitative PCR

Total RNA was extracted from 50–100 mg of liver tissue by using TRIzol reagent (Invitrogen, USA) and reverse transcribed into cDNA with a first strand reverse transcription kit (Vazyme, China), and fluorescence quantitative PCR was performed using a SYBR Green kit (Vazyme, China) at 95°C for 30 s, 95°C for 10 s and 60°C for 30 s for 40 cycles. GAPDH was used as a control, and the relative expression levels were calculated with the 2^−ΔΔCt^ method. The primers were synthesized by Sangon Biotech (Shanghai, China), and the sequences are shown in Supplementary Table 1.

### Western blotting

100 *μ*g of liver tissue was lysed with RIPA buffer containing protease and phosphatase inhibitors. The samples were centrifuged at 12 000 rpm at 4°C for 10 min, and the supernatant was collected. The protein concentration was determined with a Pierce BCA protein quantification kit (Thermo, USA). The protein (30–100 *μ*g) was subjected to SDS‒PAGE and transferred to PVDF membranes with a semidry electrophoretic transfer cell (Bio-Rad, USA). Antibodies against collagen-1, collagen-3, *α*-SMA, pMLKL and cleaved caspase 3 (CST, USA, 1:1000 dilution) were added and incubated overnight at 4°C. Then, the membrane was incubated with goat anti-rabbit antibodies (Jackson, USA, 1:4000 dilution) for 1 h at room temperature. ECL development was performed, and the relative expression of GAPDH was detected using ImageJ (National Institutes of Health, Germany).

### TUNEL

Liver slices with a thickness of 4 *μ*m were dewaxed and stained with a TUNEL kit (Vazyme, China). The procedures were performed according to the kit instructions. Green fluorescence was observed at 520 ± 20 nm. Blue fluorescence was observed at 460 nm, and the number of dead cells was counted using Image-Pro Plus software (Media Cybernetics, USA).

## Results

### Liver fibrosis was related to anti-rSjCP1412 IgG levels in the sera of advanced schistosomiasis

**Figure 1.**
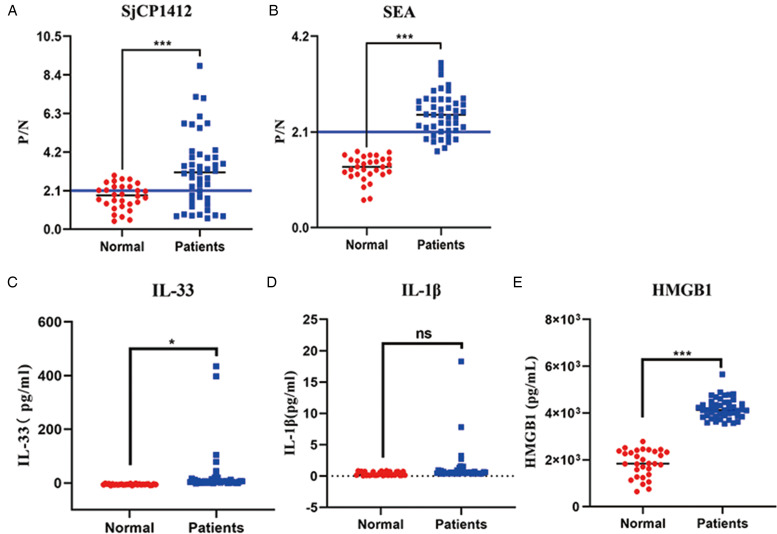
The levels of anti-rSjCP1412 IgG and DAMPs in the sera of advanced schistosomiasis and healthy patients. (a–b) Anti-rSjCP1412 and SEA IgG levels in sera; (c–e) DAMPs levels (IL-33, IL-1*β* and HMGB1) in sera. Compared with healthy controls, *P < 0.05, ***P < 0.001, ns, non-significant.

To understand the IgG levels of anti-SjCP1412 in the serum of advanced schistosomiasis, rSjCP1412 or SEA-coated ELISA was used to detect the antibody levels in the sera of patients. The results showed that the level of anti-rSjCP1412 in the sera of advanced schistosomiasis patients significantly increased (*t* = 4.258, *P* < 0.05), with a positive rate of 66.67% compared with healthy individuals. The levels of anti-SEA antibodies in the serum of advanced schistosomiasis patients were significantly increased (*t* = 68.325, *P* < 0.05), with a positive rate of 80% compared with healthy individuals (Fig. 1a, b). To further confirm that cell apoptosis and necrosis may happen in advanced schistosomiasis patients and DAMPs were produced to promote liver fibrosis in schistosomiasis, which were detected in sera of healthy individuals and advanced schistosomiasis patients using ELISA method. The results showed that the levels of IL-33 and HMGB1 in the sera of advanced schistosomiasis patients were significantly higher than those in the sera of healthy individuals (*F* = 4.55, 212.09, *P* < 0.05) (Fig. 1c–e). To determine the relationship between SjCP1412 and schistosomiasis-induced liver fibrosis, a Spearman rank correlation analysis was used to determine the relationship between serum anti-rSjCP1412 and SEA IgG levels in advanced schistosomiasis patients and the 4 factors (laminin [LN], hyaluronic acid [HA], type III procollagen [PIIIP], type IV collagen [CIV]) of liver fibrosis. The results showed that anti-rSjCP1412 antibody levels were positively correlated with LN, and the degree of the correlation was moderate (*r* = 0.388, *P* < 0.05) (Table 1). These results demonstrated that SjCP1412 may be an important egg antigen that causes schistosomiasis-induced liver fibrosis.

**Table 1.** Correlation analyses of anti-rSjCP1412 IgG level and liver fibrosis indicators in the sera of advanced schistosomiasis patients

		Laminin (LN)	Hyaluronic acid (HA)	Type III procollagen (PⅢP)	Type IV collagen (CⅣ)
Anti-rSjCP1412 IgG	*r*	0.388**	0.110	−0.198	0.240
	*P*	0.008	0.473	0.191	0.112
Anti-SEA IgG	*r*	−0.082	0.250	−0.060	0.283
	*P*	0.593	0.098	0.694	0.060

Note: *P < 0.05, **P < 0.01, ***P < 0.001.

### rSjCP1412 could induce apoptosis and necrosis in Hep G2 and LSECs *in vitro*

Hepatocytes, which are liver parenchymal cells, account for 80% of the total number of cells in the liver (Gissen and Arias, 2015). Hepatocyte death could initiate the recruitment of inflammatory cells and activate HSCs (Tu *et al*., 2015). LSECs are the main non-parenchymal cells in the liver and can maintain the quiescence of HSCs through paracrine signalling factors, such as nitric oxide (Deleve *et al*., 2008; Xie *et al*., 2012). The effect of rSjCP1412 on Hep G2 cells and LSECs was observed *in vitro*.

**Figure 2.**
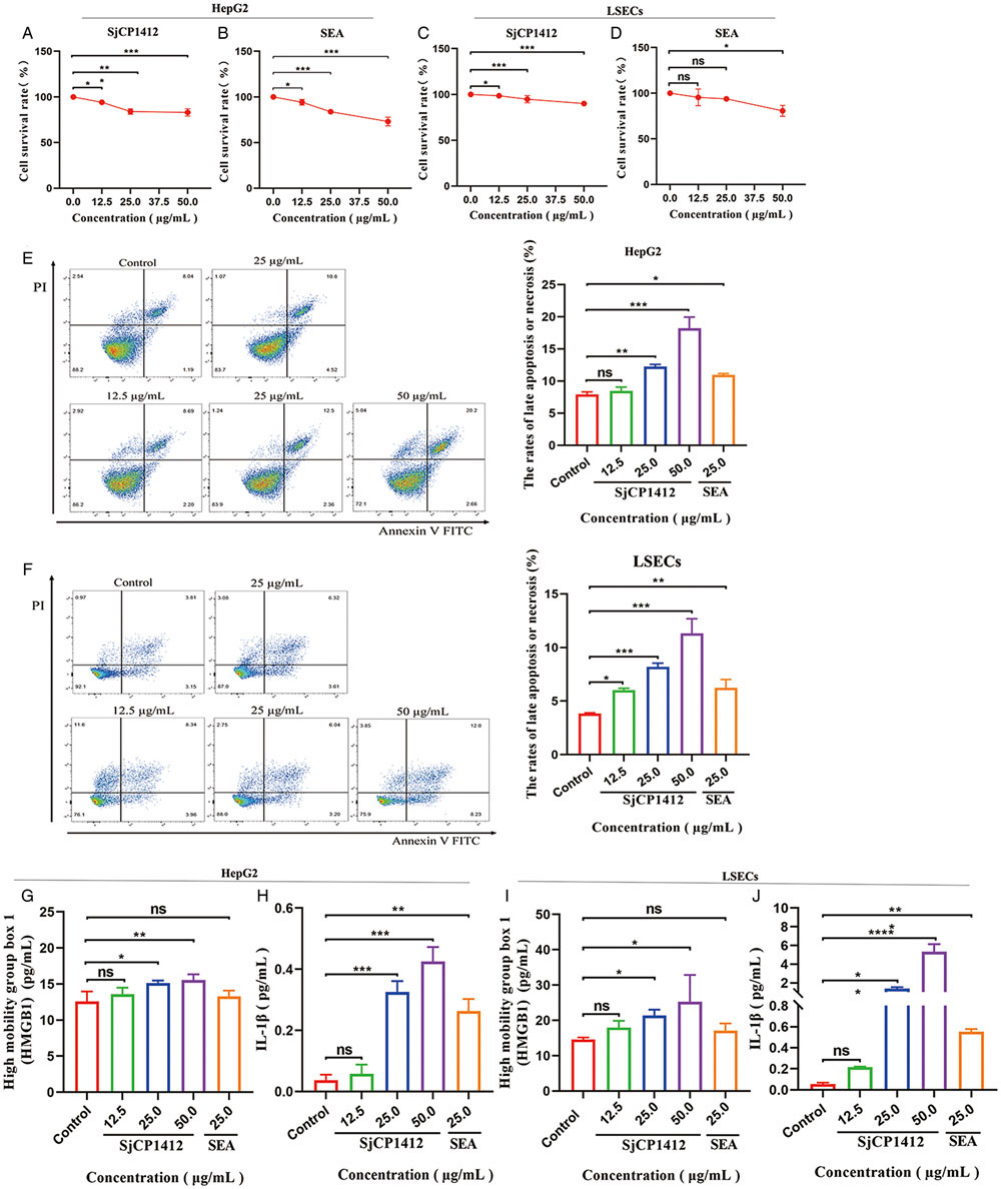
Apoptosis and necrosis in HepG2 and LSECs stimulated by rSjCP1412 *in vitro*. (a–b) Effects of rSjCP1412 and SEA on the proliferation of Hep G2 cells. (c–d) Effects of rSjCP1412 and SEA on the proliferation of LSECs. (e) rSjCP1412 induced apoptosis and necrosis in Hep G2 cells. (f) rSjCP1412 induced apoptosis and necrosis in LSECs. (g–h) IL-1*β* and HMGB1 levels in Hep G2 cells stimulated by rSjCP1412 *in vitro*. (i–j) IL-1*β* and HMGB1 levels in LSECs stimulated by rSjCP1412 *in vitro.* Compared with the control group, *P < 0.05, **P < 0.01, ***P < 0.001, ns, not significant.

The CCK-8 method was used to test the cell survival, and the results showed that compared with that in the control group, the survival rate of Hep G2 cells was significantly decreased after treatment with 12.5, 25.0 and 50.0 *μ*g mL^−1^ rSjCP1412 or SEA for 48 h (*F* = 24.178, 65.800, *P* < 0.05). The survival rate of LSECs was also significantly decreased (*F* = 16.349, 8.811, *P* < 0.05) (Fig. 2a–d). The results indicated that both SEA and rSjCP1412 proteins can significantly inhibit the proliferation of Hep G2 cells and LSECs, and the inhibitory effect gradually increased with increasing concentration.

Subsequently, flow cytometry was used to detect cell death. The results of Annexin V-FITC/PI double staining showed that compared with those in the control group, the rates of late apoptosis or necrosis (Annexin V-FITC and PI double positive) in Hep G2 cells and LSECs were significantly increased after treatment with 12.5, 25.0 and 50.0 *μ*g mL^−1^ rSjCP1412 for 48 h (*F* = 11.35, 64.90, 45.410, *P* < 0.05). The rate of late apoptosis or necrosis was dependent on the concentration of the protein. Treatment with 25 *μ*g mL^−1^ SEA resulted in apoptosis and necrosis in Hep G2 cells and LSECs, and there were statistically significant differences compared to those in the negative group (*t* = 4.486, 3.472, 4.089, *P* < 0.05) (Fig. 2e–f).

The levels of DAMPs in cell culture supernatant were tested with ELISA kits. The results showed that the levels of IL-1*β* in the culture supernatant of Hep G2 cells and LSECs were significantly increased after treatment with 12.5, 25.0 and 50.0 *μ*g mL^−1^ rSjCP1412 for 48 h (*F* = 95.311, 18.129, *P* < 0.05), and the levels of HMGB1 were significantly increased (*F* = 6.316, 3.985, *P* < 0.05). As the concentration of rSjCP1412 protein increased, the concentration of HMGB1 or IL-1*β* secreted by cell gradually increased, in a dose-dependent manner. Compared with those in the control group, 25 *μ*g mL^−1^ SEA increased IL-1*β* levels (*t* = 18.129, 40.262, *P* < 0.05). However, no increase in HMGB1 levels was observed (*t* = 1.460, 2.320, *P* > 0.05) (Fig. 2g–j).

### rSjCP1412 immunization or neutralization of the SjCP1412 protein inhibited egg granulomas and liver fibrosis in mice infected with schistosomes

**Figure 3.**
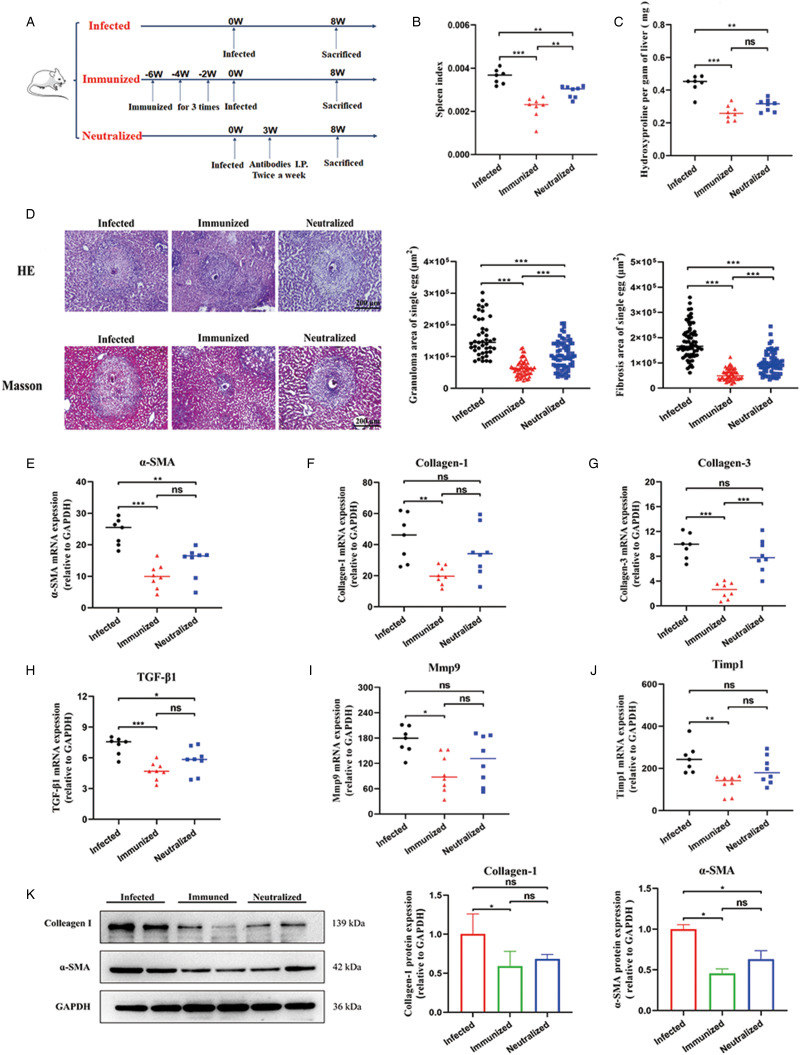
Anti-SjCP1412 immunity and antibody neutralization reduced the severity of liver lesions in mice infected with schistosomes. (a) Treatment strategies for mice in the infection group, immune group and antibody neutralization group (8 mice per group, there was 1 mouse dead in the infected group). (b) Splenic indices of schistosome-infected mice in the different groups. (c) Hydroxyproline in the livers of schistosome-infected mice in the different groups. (d) Liver egg granulomas (HE staining, scale = 200 *μ*m) and fibrosis (Masson staining, scale = 200 *μ*m) in schistosome-infected mice in the different groups and the areas of single egg granulomas and liver fibrosis. (e–j) Relative mRNA expression of *α*-SMA, collagen-1, collagen-3, TGF-*β*1, Mmp9 and Timp1 in the livers of schistosome-infected mice in the different groups. (k) The expression levels of *α*-SMA and collagen-1 in the livers of schistosome-infected mice in the different groups. Compared to the infected group, *P < 0.05, **P < 0.01, ***P < 0.001, ns, non-significant.

To further investigate the role of SjCP1412 *in vivo*, rSjCP1412 was used to immunize mice (the antibody titre reaching 1: 800 000 after 4 immunizations), or anti-rSjCP1412 antibodies (titre of 1: 200 000) were used to treat schistosome-infected mice (Fig. 3a). The liver damages of each group of mice were evaluated by measuring the SI, the content of hydroxyproline and liver pathology with HE and Masson staining. The results showed that the SI in the immune group and antibody neutralization group was significantly decreased compared to that in the infection group at 8th week post infection (*F* = 23.580, *P* < 0.05) (Fig. 3b). Compared with those in the infection group, hydroxyproline, a unique amino acid of collagen, in the immune group and the antibody neutralization group was significantly decreased (*F* = 19.932, *P* < 0.05) (Fig. 3c). Liver histopathological sections showed that the mean areas of granulomas (HE staining) and liver fibrosis (Masson staining with blue) of the single egg in the immune group and antibody neutralization group were significantly reduced compared with those in the infection group (*F* = 64.240, 113.172, *P* < 0.05) (Fig. 3d). The qRT-PCR results showed that the mRNA expression levels of related molecules in liver fibrosis, such as *α*-SMA, collagen-1, collagen-3, mmp9, timp1 and TGF-*β*1, in liver tissue were significantly decreased in the immune group compared with the infection group (*t* = 8.684, 4.973, 9.545, 4.104, 5.371 and 6.575, all *P* < 0.05) (Fig. 3e–j), indicating significant inhibition of HSCs activation in the liver. The immunoblotting results showed that the protein expression of *α*-SMA in the immune group and antibody neutralization group was decreased compared with that in the infection group, and the difference was statistically significant (*t* = 8.628, 6.584, *P* < 0.05). The protein expression of collagen-1 in the immune group was significantly decreased (*t* = 4.359, *P* < 0.05) (Fig. 3k). These results showed that rSjCP1412 immunization or neutralization of the SjCP1412 protein in mice could inhibit egg granulomas and liver fibrosis.

### rSjCP1412 immunity or neutralization of the SjCP1412 protein alleviated hepatocyte damage and DAMPs release in the mice infected with schistosome

**Figure 4.**
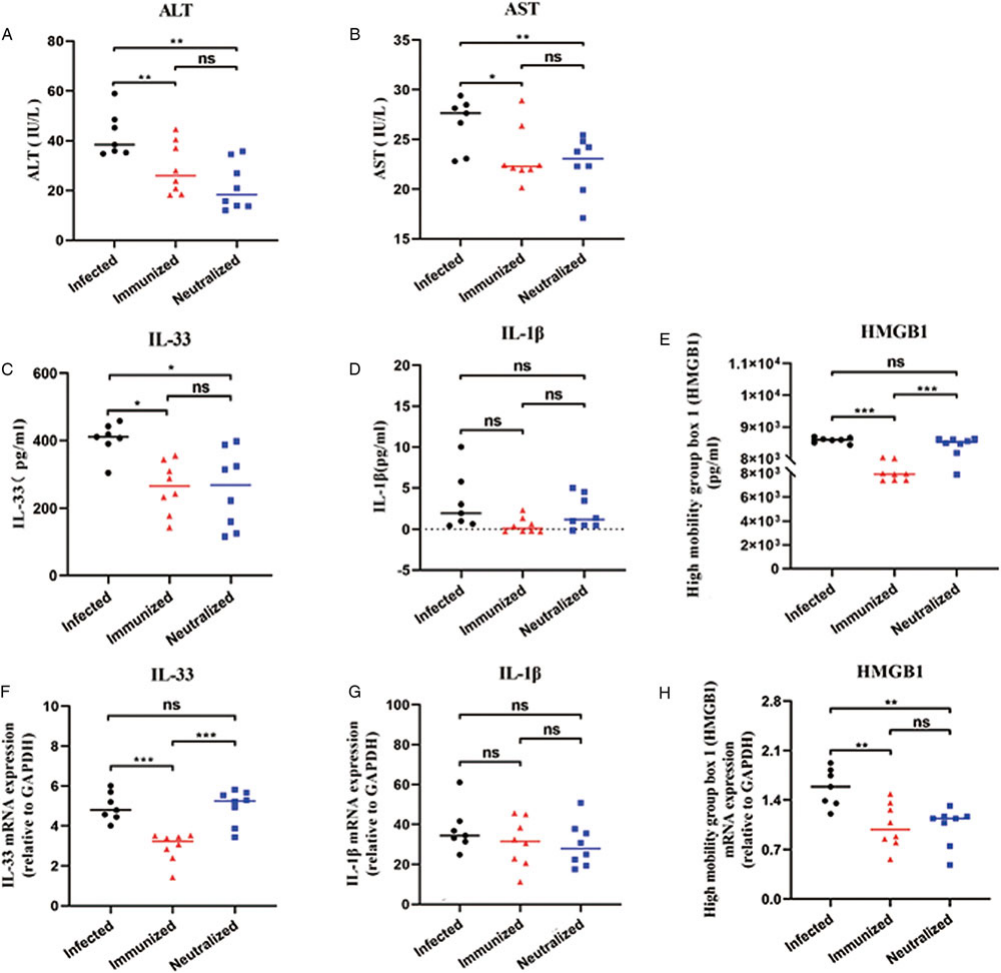
Anti-SjCP1412 immunity and antibody neutralization reduced hepatocyte damage and DAMPs release. (a) ALT level in the serum of schistosome-infected mice in the different groups. (b) AST level in the serum of schistosome-infected mice in the different groups. (c–e) Serum IL-33, IL-1*β* and HMGB1 levels of schistosome-infected mice in the different groups. (f–h) IL-33, IL-1*β* and HMGB1 mRNA levels in the livers of schistosome-infected mice in the different groups.

To determine the liver damages, the serum levels of ALT, AST and DAMPs (IL-33, IL-1*β* and HMGB1) in mice were detected with ELISA kits, respectively. Meanwhile, the mRNA expression levels of IL-33, IL-1*β* and HMGB1 in mouse liver tissue were detected by qRT-PCR. The results showed the serum levels of ALT and AST significantly reduced (*F* = 10.643, 6.244, *P* < 0.05), indicating liver damage in the immune group and antibody neutralization group alleviated (Fig. 4a, b). Serum levels of IL-33 and HMGB1 of DAMPs in immunized mice were significantly reduced (*t* = 4.270, 5.717, *P* < 0.05). Serum levels of IL-33 and HMGB1 in the antibody neutralization group showed no significant changes (*t* = 1.026, 1.944, *P* > 0.05) (Fig. 4c–e). The mRNA expression levels of IL-33 and HMGB1 in the liver tissue of immunized mice were significantly decreased (*t* = 6.133, 4.654, *P* < 0.05). There was no significant change in IL-33 mRNA levels in the antibody neutralization group (*t* = 0.320, *P* > 0.05), but HMGB1 mRNA expression was significantly decreased (*t* = 5.971, *P* < 0.05). Compared with that in the infected group, there was no significant change in the mRNA expression of IL-1*β* in the liver in the immune group and antibody neutralization group (*F* = 0.7499, *P* > 0.05) (Fig. 4f–h). These results indicated that rSjCP1412 immunization or neutralization of the SjCP1412 protein in mice infected with schistosome could reduce hepatocyte damage and DAMPs release.

### rSjCP1412 immunity or neutralization of the SjCP1412 protein alleviated apoptosis and necroptosis in the livers of mice infected with schistosomes

**Figure 5.**
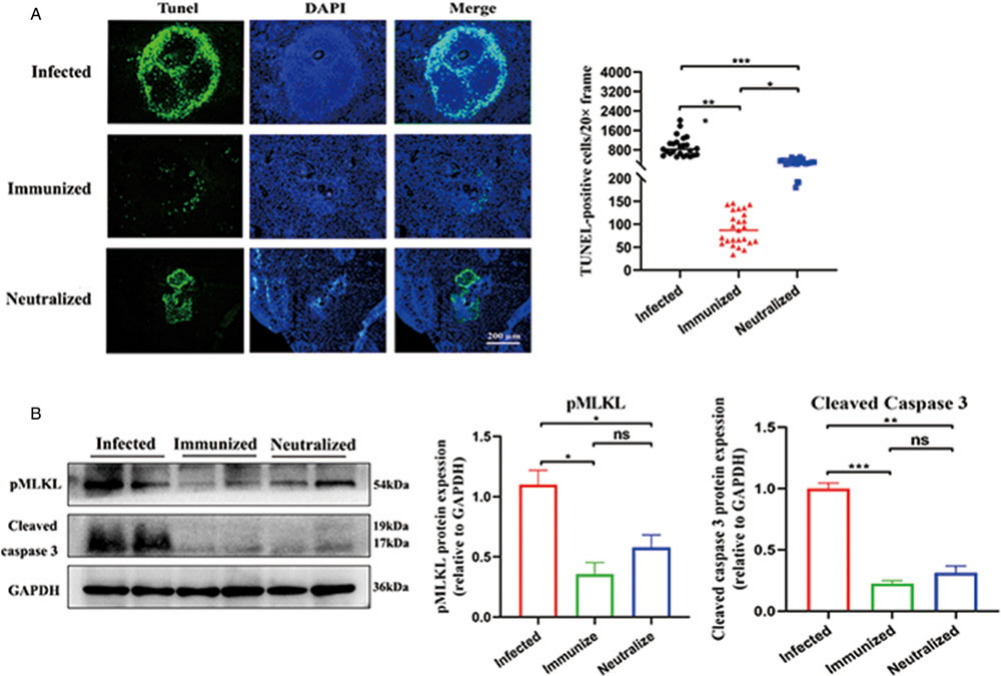
Anti-SjCP1412 immunity and antibody neutralization reduced liver damage and DAMPs levels *via* apoptosis and necroptosis. (a) TUNEL staining of the liver tissues of schistosome-infected mice in the different groups. Apoptotic cells were stained green, and nuclei were stained blue by DAPI staining solution (scale = 200 *μ*m). (b) Changes in the expression levels of relevant cell death proteins (pMLKL and cleaved caspase 3) in the liver tissues of schistosome-infected mice in the different groups. Compared to the infected group, *P < 0.05, **P < 0.01, ***P < 0.001, ns, non-significant.

To further understand the cell apoptosis and necrosis in the liver tissues of the immune and antibody neutralizing groups infected with schistosome, cell apoptosis in the liver tissues of mice were detected by TdT-mediated dUTP Nick-End Labeling (TUNEL) staining, and the expression levels of related apoptotic and necrotic proteins were detected by immunoblotting. TUNEL staining showed that the number of apoptotic cells in the liver in the immune group and antibody neutralization group was significantly reduced compared with that in the infection group (*F* = 78.34, *P* < 0.05), and apoptotic cells were mainly distributed around the egg granuloma (Fig. 5a). The immunoblotting results showed that the expression levels of p-MLKL, which is a marker of necroptosis, and cleaved caspase 3, which is a marker of apoptosis, were decreased in the liver in the immune and antibody neutralization groups compared with the infection group (*F* = 25.58, 196.6, *P* < 0.05) (Fig. 5b). These results proved that rSjCP1412 immunization or antibody neutralization of SjCP1412 activity inhibited apoptosis and necroptosis around the eggs, which decreased inflammation levels, thereby reducing egg granuloma inflammatory reactions and liver fibrosis.

## Discussion

The main pathological manifestations of schistosomiasis are granuloma lesions, periportal fibrosis and subsequent portal hypertension caused by the SEA of eggs deposited in the liver, which causes serious harm to health (Pearce and MacDonald, 2002; Wynn *et al*., 2004). Identifying the component of SEA involved in the inflammatory response of egg granulomas can help clarify the molecular mechanism of liver fibrosis, thus providing a basis for the prevention and treatment of schistosomiasis-induced liver fibrosis. In this study, the recombinant egg ribonuclease SjCP1412 of *S. japonicum* has RNase activity and was successfully prepared (Supplementary Fig. 1). Similar to omega-1 in *S. mansoni* (Fitzsimmons *et al*., 2005), SjCP1412 is a member of the RNase T2 family and is an exocrine protein that is specifically expressed in eggs (Ke *et al*., 2017). qPCR and immunoblotting analysis showed that SjCP1412 was secreted specifically by eggs, was not expressed in cercariae or adults in our previous study (Ke *et al*., 2017) and may be involved in the formation of egg granulomas and liver fibrosis. We further demonstrate that the anti-SjCP1412 IgG in the serum of advanced schistosomiasis was moderately correlated with liver fibrosis by analysing the serum epidemiology of the population. SjCP1412 protein had hepatotoxicity inducing cell apoptosis and necrosis and the release of pro-inflammatory DAMPs *in vitro*. Recombinant SjCP1412 protein was used to immunize mice, or anti-rSjCP1412 antibodies were intraperitoneally injected to neutralize SjCP1412 activity in mice, which significantly reduced cell apoptosis and necroptosis in liver tissue, thereby reducing the inflammatory and liver fibrosis.

First of all, to understand the IgG levels of anti-SjCP1412 in the serum of advanced schistosomiasis patients, whose main clinical symptom is liver fibrosis (LoVerde, 2019), rSjCP1412 or SEA were used to examine anti-SjCP1412 or anti-SEA IgG in the serum. There were high levels of anti-SjCP1412 and anti-SEA IgG in the sera of advanced schistosomiasis patients, and positive rates were 66.67 and 80%, respectively. This finding indicated that there was continuous release of egg antigens in these advanced schistosomiasis patients, which may be related to the continuous progression of liver disease in these patients. Correlation analysis showed that anti-rSjCP1412 IgG was moderately correlated with LN, while the level of anti-SEA IgG was not related to the 4 factors of liver fibrosis, indicating that the secreted egg protein SjCP1412 may be related to the development of advanced schistosomiasis.

By detecting the levels of DAMPs in the sera of healthy and advanced schistosomiasis patients, it was found that the levels of HMGB1 and IL-33 in the sera of advanced schistosomiasis patients were significantly higher than those of healthy patients, indicating that DAMPs may be closely correlated with liver fibrosis of schistosomiasis. DAMPs molecules, often released by apoptotic or necrotic cells, are important inducers of inflammation (Garg *et al*., 2012; Kaczmarek *et al*., 2013). In addition, SjCP1412 is a ribonuclease that degrades RNA, which may cause cell death by degrading cellular RNA. Therefore, HepG2 cells or LSECs death induced by rSjCP1412 were detected *in vitro.* The CCK-8 and flow cytometry results suggested that SjCP1412 had hepatotoxicity as omega-1 in *S. mansoni* (Doenhoff *et al*., 1981; Dunne *et al*., 1991), which could induce cell apoptosis and necrosis releasing of pro-inflammatory DAMPs and may play an important role in liver lesions caused by schistosomiasis.

To verify this hypothesis, rSjCP1412 was used to immunize mice, and purified anti-SjCP1412 IgG was used to treat mice infected with *S. japonicum* for 4 weeks. The results showed that rSjCP1412 immunization or antibody neutralization of SjCP1412 activity could significantly reduce egg granulomas and liver fibrosis. Furthermore, we found that blocking SjCP1412 could reduce the level of DAMPs, including HMGB1 and IL-33, released from apoptotic or necrotic cells, confirming our hypothesis. HMGB1 can be released from dead cells or secreted from immune cells, which promotes the secretion of inflammatory cytokines (Rauvala and Rouhiainen, 2007). HMGB1 promotes the proliferation and activation of HSCs to promote liver fibrosis during disease progression, and the high level of HMGB1 at the injury site recruits fibroblasts, endothelial cells and smooth muscle cells to repair tissue damage (Vicentino *et al*., 2018; Chen *et al*., 2021). HMGB1 inhibitors significantly inhibit the progression of liver fibrosis, indicating that HMGB1 plays an important role in liver fibrosis. IL-33 belongs to the IL-1 family, is typically present in the nucleus and is released into the extracellular space during tissue damage (Bai *et al*., 2021). IL-33 plays an important role in the immune response to schistosome infection, and the level of IL-33 is positively correlated with the progression of egg granulomas and liver fibrosis (Zhang *et al*., 2021). IL-33 induces M2-type polarization in macrophages and the release of IL-5 and IL-13 to promote liver fibrosis (Li *et al*., 2019). IL-33 promotes the recruitment of the second group of congenital lymphocytes (ILC2s) in the liver, and ILC2s secrete IL-13 to promote the development of liver fibrosis (He *et al*., 2018). IL-33 helps to maintain the Th2 immune microenvironment of the liver in mice infected with schistosomes (Li *et al*., 2019; Zhang *et al*., 2021), which plays a critical role in the hepatic immunopathology of schistosomiasis (Zheng *et al*., 2020).

This study also showed that compared with that in the infection group, liver pathological damage in the antibody neutralization group was significantly reduced, but the degree of the reduction was lower than that in the SjCP1412 immunization group, which may be related to the lower titre of the neutralizing antibody injected compared to the level of the anti-SjCP1412 antibody in immunized mouse serum. The levels of anti-rSjCP1412 polyclonal antibodies were 1: 200 000, while the antibody levels in rSjCP1412-immunized mice reached 1: 800 000. The neutralization effects of the anti-rSjCP1412 antibody may be influenced by the antibody titre, quantity and frequency of injection. In addition, neutralizing antibody injection may induce anti-antibody production, leading to a weakened effect of the antibody against SjCP1412, which need to be further studied. Further exploration of the optimal strategy for antibody treatment would enhance antibody neutralization to alleviate the egg granulomas and fibrosis during schistosomiasis.

## Conclusions

To conclude, our findings demonstrate that the SjCP1412 protein could promote egg granulomas and liver fibrosis by inducing apoptosis and necrosis *via* DAMPs *in vivo* and *in vitro*. Blocking SjCP1412 could inhibit its proapoptotic and necrotic effects and alleviate liver fibrosis, which may be a potential strategy for inhibiting schistosomiasis-induced liver fibrosis. Consistently, we found that SjCP1412 protein played a crucial role in promoting liver fibrosis during schistosomiasis through inducing an inflammatory reaction involving DAMPs mediated by apoptosis and necroptosis. This study further clarified the mechanism of the formation of liver fibrosis and provided ideas for exploring new treatment methods for liver fibrosis.

## Supporting information

Li et al. supplementary material 1 Li et al. supplementary material

Li et al. supplementary material 2 Li et al. supplementary material

Li et al. supplementary material 3 Li et al. supplementary material

## Data Availability

The datasets generated during and/or analysed during the current study are available from the corresponding author on reasonable request.

## Supplementary material

The supplementary material for this article can be found at https://doi.org/10.1017/S0031182023001361.
