# Supplementary material for: The egg ribonuclease SjCP1412 accelerates liver fibrosis caused by Schistosoma japonicum infection involving damage-associated molecular patterns (DAMPs)
Source: Parasitology. 2023 Dec 18;151(3):260–70. doi: 10.1017/S0031182023001361 (PMC11007278; doi:10.1017/S0031182023001361)
Supplement: Li et al. supplementary material 1 — Li et al. supplementary material [file S0031182023001361sup001.docx]

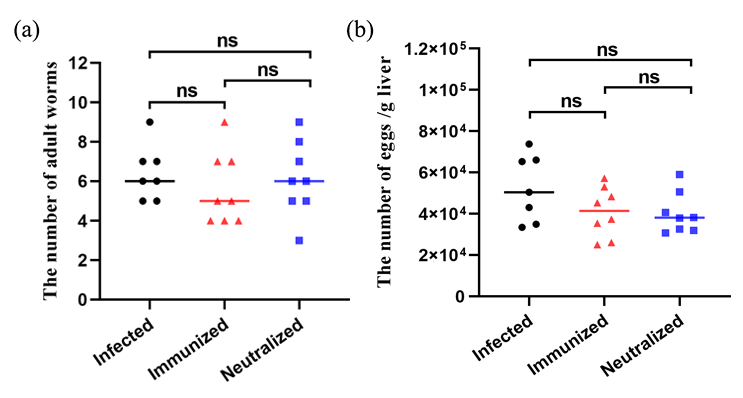


**Supplementary material figure 2.** The number of adult worms and eggs in mice infected with schistosomes (a) The number of adult worms in the different groups. (b) The number of eggs per g of liver in the different groups. Compared to the infected group, ns: nonsignificant.
