# Supplementary material for: The egg ribonuclease SjCP1412 accelerates liver fibrosis caused by Schistosoma japonicum infection involving damage-associated molecular patterns (DAMPs)
Source: Parasitology. 2023 Dec 18;151(3):260–70. doi: 10.1017/S0031182023001361 (PMC11007278; doi:10.1017/S0031182023001361)
Supplement: Li et al. supplementary material 2 — Li et al. supplementary material [file S0031182023001361sup002.docx]

**
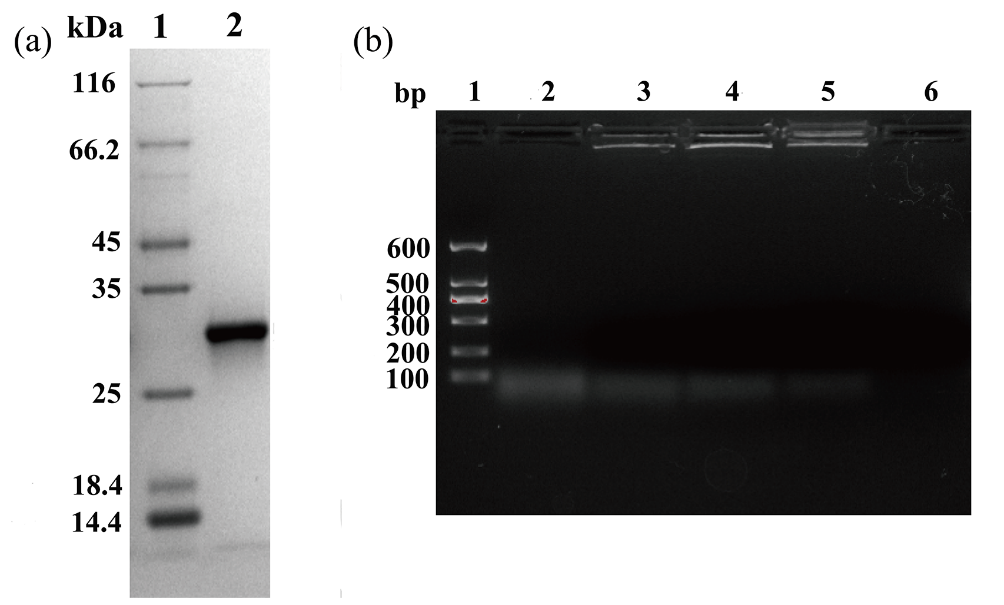
**

**Supplementary material figure 1.** rSjCP1412 protein expression and functional analysis of RNase activity (a) SjCP1412 protein expression. (1) The protein molecular weight standard; (2) the purification rSjCP1412. (b) RNase activity of rSjCP1412. (1) DNA molecular weight standard; (2) undigested yeast RNA (negative control); (3) yeast RNA digested by 10 μg of rSjCP1412; (4) yeast RNA digested by 20 μg of rSjCP1412; (5) yeast RNA digested by 40 μg of rSjCP1412; (6) yeast RNA digested by a commercial RNase A (positive control).
