# Supplementary material for: The egg ribonuclease SjCP1412 accelerates liver fibrosis caused by Schistosoma japonicum infection involving damage-associated molecular patterns (DAMPs)
Source: Parasitology. 2023 Dec 18;151(3):260–70. doi: 10.1017/S0031182023001361 (PMC11007278; doi:10.1017/S0031182023001361)
Supplement: Li et al. supplementary material 3 — Li et al. supplementary material [file S0031182023001361sup003.docx]

**Supplementary material table 1.** qPCR primer sequences

|  | sequences |
| --- | --- |
| *Collagen Ⅰ* | Forward: CCAAGAAGACATCCCTGAAGTCA  Reverse: TGCACGTCATCGCACACA |
| *Collagen Ⅲ* | Forward: CTGTAACATGGAAACTGGGGAAA  Reverse: CCATAGCTGAACTGAAAACCACC |
| *α-SMA* | Forward: CCCAGACATCAGGGAGTAATGG  Reverse: TCTATCGGATACTTCAGCGTCA |
| *TGF-β1* | Forward: CACTGGAGTTGTACGGCAGTG  Reverse: AGAGCAGTGAGCGCTGAATC |
| *Mmp-9* | Forward: CGCCTTGGTGTAGCACAACA  Reverse: ACAGGGTTTGCCTTCTCCGTT |
| *Timp-1* | Forward: CGAGACCACCTTATACCAGCG  Reverse: ATGACTGGGGTGTAGGCGTA |
| *IL-33* | Forward: GATGGGAAGAAGGTG ATGGTG  Reverse: TTG TGAAGGACGAAGAAGGC |
| *IL-1β* | Forward: GGAAGCAGCCCTTCATCTTT  Reverse: TGGCAACTGTTCCTGAACTC |
| *HMGB1* | Forward：ATATGGCAAAAGCGGACAAG  Reverse：AGGCCAGGATGTTCTCCTTT |
| *GAPDH* | Forward: AACTTTGGCATTGTGGAAGG  Reverse: CCCTGTTGCTGTAGCCGTAT |
